# Endoscopic Ultrasound-Guided Celiac Plexus Block-Induced Peripancreatic Abscess Requiring Transgastric Drainage

**DOI:** 10.14309/crj.0000000000001856

**Published:** 2025-10-16

**Authors:** Sanjeevani Tomar, Sagar Pathak, Saurabh Chandan, Abdullah Abbasi, Maham Hayat, Natalie D. Cosgrove, Kambiz S. Kadkhodayan, Dennis Yang, Deepanshu Jain, Muhammad K. Hasan, Mustafa A. Arain

**Affiliations:** 1 Department of Gastroenterology and Hepatology, AdventHealth Orlando, FL; 2 Center of Interventional Endoscopy, AdventHealth Orlando, FL

**Keywords:** chronic pancreatitis, endoscopic ultrasound, celiac plexus block, abscess, pancreatic pseudocyst, endoscopic drainage

## Abstract

Endoscopic ultrasound-guided celiac plexus block (CPB) is a widely used technique for managing refractory abdominal pain in patients with chronic pancreatitis. Although generally safe, adverse events such as infection and abscess formation are important considerations in the risk profile of the procedure. We present a case of a 49-year-old woman with a history of chronic pancreatitis who developed an abscess at the site of a CPB that failed medical therapy and required endoscopic ultrasound-guided transgastric drainage. This case highlights the need for appropriate clinical application of CPB and the importance of recognition and management of postprocedural adverse events.

## INTRODUCTION

Chronic pancreatitis is a debilitating condition often associated with severe abdominal pain that is refractory to medical therapy. Endoscopic ultrasound (EUS)-guided celiac plexus block (CPB) is used as a minimally invasive intervention for refractory pain associated with chronic pancreatitis.^1–3^ However, postprocedural infectious adverse events, such as abscess formation, are poorly characterized in the literature.^4,5^ We describe a case of CPB related localized abscess adjacent to the celiac axis necessitating EUS-guided transmural drainage.

## CASE REPORT

A 49-year-old woman with a history of recurrent acute pancreatitis and multiple comorbidities presented to an outside hospital with severe, progressively worsening upper abdominal pain associated with nausea and vomiting for 4 days. She had a history of alcohol use but had been sober for several years. Her surgical history included cholecystectomy for gallstone disease, sleeve gastrectomy, and subsequent conversion to a Roux-en-Y gastric bypass (RYGB) for morbid obesity. She had previously undergone an endoscopic ultrasound-directed transgastric endoscopic retrograde (EDGE) with biliary and pancreatic sphincterotomy to achieve biliary access in the setting of RYGB anatomy, performed for evaluation and management of suspected pancreatobiliary pathology contributing to recurrent symptoms. This provided symptom relief for 9 months before her pain recurred despite normal pancreatic enzyme levels. Following recurrence, she underwent 3 CPBs over a 5-month period: the first 5 months before presentation, the second 3 months prior, and the third 2 weeks before admission. According to available records, each block involved injection of 20 mL of 0.25% bupivacaine mixed with 80 mg of triamcinolone in the region of the celiac axis. Prophylactic antibiotics were not administered before the CPBs. Her medical history was also significant for possible early primary biliary cholangitis, hypertension, hyperlipidemia, and hypothyroidism. She had no history of immunosuppression, steroid use beyond the triamcinolone in the plexus block, or other conditions predisposing to infection.

**Figure 1.**
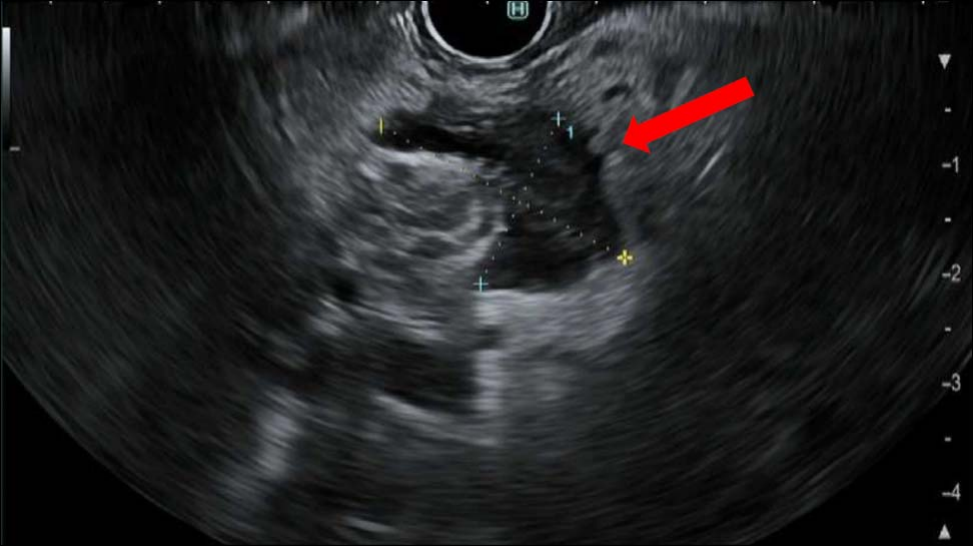
Endoscopic ultrasound showing an irregular fluid collection measuring 25 × 17 mm adjacent to the celiac axis.

On admission, laboratory tests revealed a white blood cell count of 14.7 × 10^3^/µL, hemoglobin of 13.6 g/dL, platelet count of 268 × 10^3^/µL, lipase of 8 U/L, and a normal hepatic panel. Abdominal and pelvic computed tomography (CT) with intravenous contrast revealed a concern for contained perforation at the gastrojejunal anastomosis with a bilobed fluid collection, prompting transfer to our facility for further evaluation. On endoscopy, she had standard RYGB anatomy without any mucosal abnormality and on EUS a 25 by 17-mm anechoic fluid collection with dense hyperechoic foci concerning for an abscess was seen adjacent to the celiac axis (Figure 1). A 19-gauge needle was used to aspirate 4 cc of purulent fluid. Cultures grew 3+ *Streptococcus constellatus*, whereas fungal and anaerobic cultures were negative. The abscess was suspected to be a sequela of the recent CPB procedures. She was treated with intravenous piperacillin-tazobactam, and a follow-up abdominal CT without intravenous contrast the next day showed resolution of the fluid collection. Given clinical improvement, she was discharged with a 3-day course of amoxicillin/clavulanic acid per infectious disease recommendations, with a plan to follow-up in our pancreas clinic.

**Figure 2.**
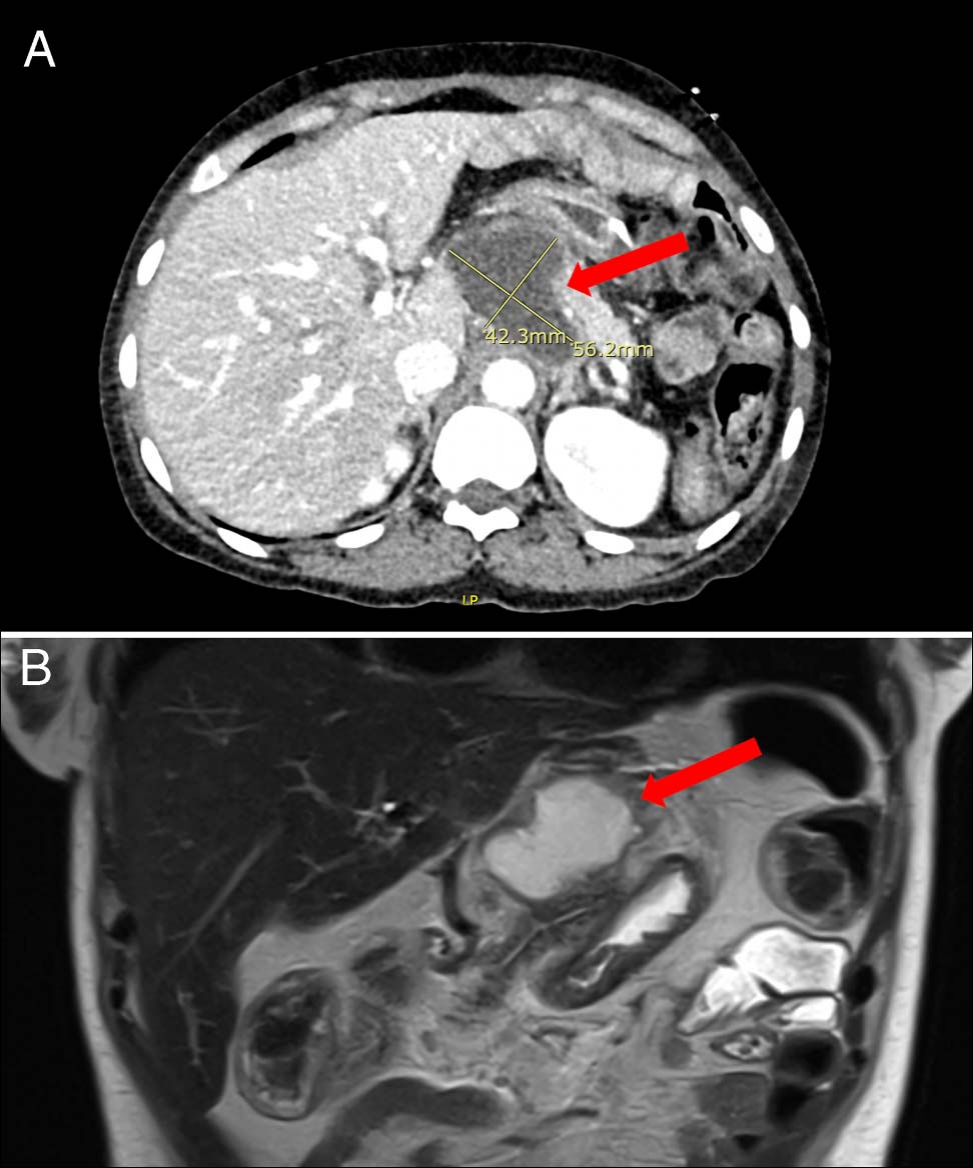
(A) CT scan showing a peripancreatic fluid collection measuring 5.6 × 4.2 × 3.3 cm with mild peripancreatic fat stranding. (B) Magnetic Resonance Imaging/Magnetic Resonance Cholangiopancreatography confirming a well-defined peripancreatic fluid collection measuring 6 × 4.2 × 4.8 cm adjacent to the celiac axis.

**Figure 3.**
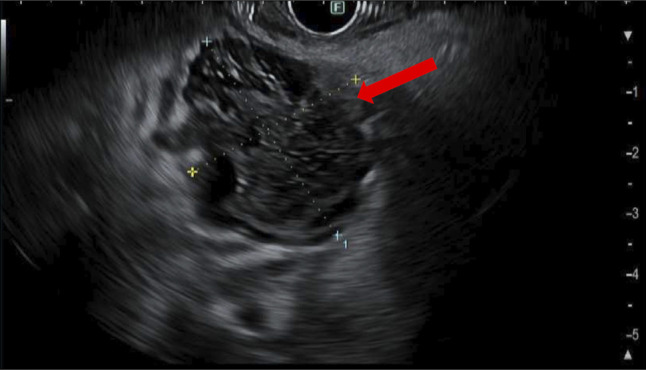
Endoscopic ultrasound showing a 38 × 31-mm peripancreatic fluid collection.

**Figure 4.**
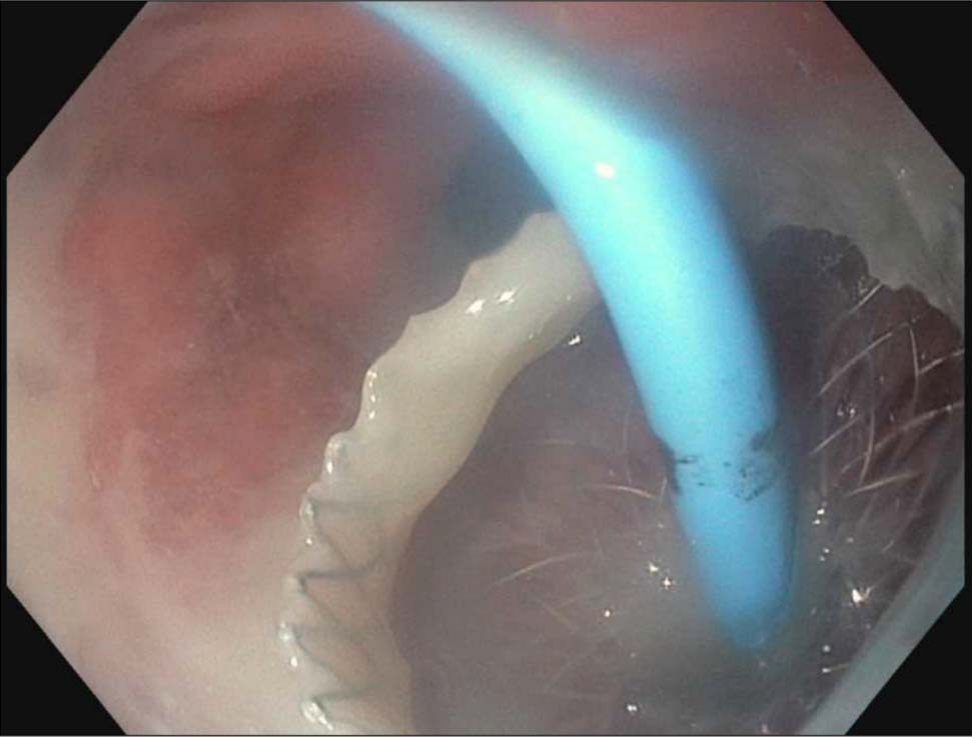
Lumen-apposing metal stent (LAMS) and double pigtail stent via the gastric pouch, with successful drainage of purulent material.

One month later, the patient re-presented with recurrent abdominal pain. Laboratory testing revealed a leukocytosis with a white blood cell count of 14.9 × 10^3^/μL, hemoglobin of 12.8 g/dL, platelet count of 379 × 10^3^/μL, lipase of 9 U/L, and a normal hepatic panel. A repeat abdominal and pelvic CT with intravenous contrast revealed a recurrent fluid collection measuring 5.6 × 4.2 × 3.3 cm with mild peripancreatic fat stranding adjacent to the celiac axis (Figure 2). Magnetic Resonance Imaging/Magnetic Resonance Cholangiopancreatography without intravenous contrast confirmed a well-defined peripancreatic fluid collection measuring 6 × 4.2 × 4.8 cm (Figure 2). Blood cultures grew *Streptococcus anginosus*, and the patient was started on ceftriaxone. EUS demonstrated a 38 × 31-mm peripancreatic fluid collection, which was drained using a 10 × 10-mm lumen-apposing metal stent and a 7 Fr × 3-cm double-pigtail coaxial plastic stent placed via the gastric pouch (Figure 3). Drainage yielded purulent material (Figure 4).

After procedure, the patient's symptoms improved. She was managed with short-term narcotics during hospitalization and prescribed pregabalin for chronic pain. Follow-up imaging confirmed resolution of the abscess, and the stents were removed via upper endoscopy 4 weeks later.

## DISCUSSION

Although EUS-guided CPB is generally well tolerated, infectious adverse events such as abscess formation at the injection site can occur.^6^ This case underscores the potential for CPB to precipitate localized inflammation and infections, including abscess formation, which may require additional interventions for adequate drainage. Proposed mechanisms include bacterial translocation during injection, hematogenous spread, or direct contamination. Given the delayed onset of symptoms post-CPB, clinical vigilance is necessary for early detection and management.^2,7^

Although the fluid collection was initially suspected on CT to represent a complication of the prior EDGE procedure given its proximity to the gastrojejunal anastomosis, the timing of onset and location adjacent to the celiac axis were more consistent with a delayed sequela of the recent CPB. Although altered anatomy and previous interventions can increase infection risk, in this case the peripancreatic abscess developed shortly after the most recent plexus block, making this intervention the more likely source than the prior EDGE procedure.

The reported rate of abscess formation following EUS-guided CPB is approximately 1%.^8^ Although rare, this risk must be considered, especially in patients with predisposing conditions. In this case, chronic pancreatitis likely represented the underlying risk factor predisposing to abscess formation. Chronic pancreatitis is associated with local tissue necrosis, impaired mucosal immunity, and ductal disruption, all of which may facilitate bacterial translocation and infection following CPB.

EUS-guided CPB remains a questionable tool for pain management in chronic pancreatitis as studies have shown variable efficacy, with some patients deriving limited benefit.^5,9^ In this case, the patient underwent 3 CPBs in relatively short succession, with procedures being performed 3 and then 2 months apart. The frequency of these interventions highlights the importance of carefully assessing the response to CPB. Given its limited efficacy in a significant number of patients, and the potential for adverse events, repeat CPBs should be used judiciously, allowing sufficient time to assess for a sustained pain response and reassessment before considering additional procedures. Transient or permanent nerve injury resulting in diarrhea, hypotension and rarely, more severe injury such as paraplegia are recognized adverse events related to CPB. During discussions regarding the procedure, patients should be made aware of the possibility of infection and abscess formation. This case highlights the importance of early imaging, diagnostic aspiration, and, if necessary, endoscopic drainage in managing such adverse events. Increased awareness of this potential adverse event may help improve post-CPB monitoring protocols and early intervention strategies.

Management of post-CPB abscess formation requires a multimodal approach.^4,10^ In this patient, there was a suspicion of infection based on the clinical presentation and imaging at the time of her initial presentation to our hospital. However, the collection was deemed too small for transgastric drainage and was therefore initially aspirated and the patient was treated with antibiotics. She later re-presented with a larger, walled off collection and was treated with EUS-guided transmural drainage resulting in symptomatic relief and resolution of the abscess. Antibiotics and pain management played an adjunctive role in ensuring a favorable outcome. The management of pain symptoms associated with chronic pancreatitis continues to be a major challenge in clinical practice.^11^ Patients require a multidisciplinary approach with judicious use of interventional strategies such as CPB, given their limited efficacy for pain relief in the long-term, and associated procedure related adverse events.

## DISCLOSURES

Author contributions: S. Tomar: literature review, drafting and revising the manuscript; S. Pathak: edited the manuscript and revised for intellectual content; S. Chandan, A. Abbasi, M. Hayat, ND Cosgrove, D. Yang, D. Jain, MK Hasan: revised the article for intellectual content; MA Arain: provided the images, critically reviewed the article, and is the article guarantor.

Financial disclosure: S. Tomar, S. Pathak, S. Chandan, A. Abbasi, M. Hayat: None. ND Cosgrove: consultant for Cook Medical. KS Kadkhodayan: None. D. Yang: consultant for Boston Scientific, Fujifilm, Olympus, Medtronic, Microtech, 3D-Matrix, and Neptune Medical. Received research support from 3D-Matrix and Microtech. D. Jain: None. MK Hasan: consultant for Olympus, Boston Scientific, Microtech, Medtronic, and Neptune Medical. MA. Arain: consultant for Boston Scientific, Olympus, Cook Medical, and Medtronic. MA. Arain is the article guarantor.

Informed consent was obtained for this case report.

## ABBREVIATIONS:

µL: microliter
CPB: celiac plexus block
CT: computed tomography
EDGE: endoscopic ultrasound-directed transgastric ERCP
ERCP: endoscopic retrograde cholangiopancreatography
EUS: endoscopic ultrasound
Fr: French (catheter/stent size unit)
g/dL: grams per deciliter
LAMS: lumen-apposing metal stent
mm: millimeter
MRCP: magnetic resonance cholangiopancreatography
MRI: magnetic resonance imaging
RYGB: Roux-en-Y gastric bypass
U/L: units per liter
